# Payments for Environmental Services in a Policymix: Spatial and Temporal Articulation in Mexico

**DOI:** 10.1371/journal.pone.0152514

**Published:** 2016-04-06

**Authors:** Driss Ezzine-de-Blas, Céline Dutilly, José-Alberto Lara-Pulido, Gwenolé Le Velly, Alejando Guevara-Sanginés

**Affiliations:** 1 CIRAD (Centre de Coopération Internationale en Recherche Agronomique pour le Développement), Montpellier, France; 2 Universidad Iberoamericana del Distrito Federal, Mexico City, Mexico; 3 CERDI (CERDI Centre d'Etudes et de Recherches sur le Développement International), Auvergne University, Clermont Ferrand, France; University of Vermont, UNITED STATES

## Abstract

Government based Payments for Ecosystem Services (PES) have been criticized for not maximizing environmental effectiveness through appropriate targeting, while instead prioritizing social side-objectives. In Mexico, existing literature on how the *Payments for Ecosystem Services-Hydrological program* (PSA-H) has targeted deforestation and forest degradation shows that both the process of identifying the eligible areas and the choice of the selection criteria for enrolling forest parcels have been under the influence of competing agendas. In the present paper we study the influence of the PSA-H multi-level governance on the environmental effectiveness of the program–the degree to which forest at high risk of deforestation is enrolled- building from a “policyscape” framework. In particular, we combine governance analysis with two distinct applications of the policyscape framework: First, at national level we assess the functional overlap between the PSA-H and other environmental and rural programs with regard to the risk of deforestation. Second, at regional level in the states of Chiapas and Yucatan, we describe the changing policy agenda and the role of technical intermediaries in defining the temporal spatialization of the PSA-H eligible and enrolled areas with regard to key socio-economic criteria. We find that, although at national level the PSA-H program has been described as coping with both social and environmental indicators thanks to successful adaptive management, our analysis show that PSA-H is mainly found in communities where deforestation risk is low and in combination with other environmental programs (protected areas and forest management programs). Such inertia is reinforced at regional level as a result of the eligible areas’ characteristics and the behaviour of technical intermediaries, which seek to minimise transaction costs and sources of uncertainty. Our project-specific analysis shows the importance of integrating the governance of a program in the policyscape framework as a way to better systematize complex interactions at different spatial and institutional scales between policies and landscape characteristics.

## Introduction

Mexico has been one of the pioneer Latin American countries to have implemented a nation-wide Payment for Ecosystem Services (PES) program, here referred as the *Payment for Ecosystem Services-Hydrological program* (PSA-H), to protect critical forests for water provision and regulation services [1]. The program started in 2003 and has been successful in incorporating a large area of diverse type of forests–more than 2 million hectares of forests from conifer to lowland rainforests have been enrolled since 2003. Payments are granted for 5 years on a yearly basis to forest communities after the signature of a contract between community elected leaders and the Mexico’s National Forestry Commission (CONAFOR). Forest communities in Mexico are composed by *ejidos* (collective lands granted to modern settlers) and traditional communities: We use the term communities to signify both. Private owners can also be contracted but represent a minority of the total number of beneficiaries. So far, PSA-H impact evaluation literature has focussed on the impact of on forest cover, with current findings estimating that the program helped to reduce the expected land-cover loss by 41–50% while also generating small but positive poverty alleviation [2,3]. When it comes to the capacity of the program to target both environmental and social objectives, which stand as the main institutional priorities of the program, existing evidence shows mixed results. While some studies argue that CONAFOR has succeeded in progressively enrolling forest parcels with both a higher deforestation risk and level of marginality (a Mexican proxy for poverty, http://www.conapo.gob.mx/es/CONAPO/Indices_de_Marginacion_Publicaciones) as a result of an adaptive management strategy [4], more recent studies indicate that environmental benefits are higher where poverty is slow and vice versa [3]. The deforestation risk is a spatially explicit indicator available at national level and developed in 2010 by the National Institute for the Ecology and Climate Change (INECC) to estimate the probability of a forest pixel to be deforested [4].Overall, the program has been successful in enrolling and protecting large tracts of forest, but conclusive evidence is still lacking on the programme’s efficacy at targeting both forest at risk and poor populations.

Achieving two targets with only one instrument is a policy problem sensitive to the governance dynamics of program implementation and to the social-ecological characteristics of the landscape [5]. A mix of policies (i.e. a policymix) is needed when–alike rural Mexico- there are ‘multiple externalities or externalities occurring together with imperfect property rights, market power, unobservable behaviour, or imperfect information’ [6]. Environmental policies account from command-and-control (protected areas), to economic incentives in form of cash transfers (payments for ecosystem services) and capacity building (forest management support, community enterprises) among others. The spatial expression of such a policymix can be defined as a *policyscape* [7]. Since the risk of deforestation and marginality levels are spatially explicit and heterogeneously distributed, the capacity of a policymix to achieve both targets depends on the degree to which it aligns with them spatially. Moreover, for this alignment to happen, coordination at different governance scales must happen in accordance with adaptive management principles [8,9].

Indeed, the geographical distribution of a policy depends on its governance dynamics, especially in countries like Mexico where targeting is politically sensitive and dependant on the negotiation power of the public and private actors involved [10]. Although some studies have underscored the way federal governance has influenced the design and effectiveness of PES programs [4,11], current research has overlooked its implications in terms of a policyscape. In addition, regional governance dynamics can also determine where a program will come into action. In particular, the role of technical intermediaries in choosing what forests to enroll remains largely understudied. Technical intermediaries can have very different interests–e.g. when comparing private profit intermediaries with NGOs—and guide the spatialization of PES programs based on different socio-economic and political rationales [12,13].

In this paper, we confront governance dynamics at federal (national) and regional levels with the spatial distribution of the PAS-H and other environmental programs in order to explain the links between the multi-level governance of the programme and its capacity to target both deforestation and poverty objectives. We build a conceptual framework that combines governance analysis along two distinct policyscape applications: (i) First, at national level we compare the outcomes of federal stakeholder negotiations where the selection criteria to enrol forest parcels were decided with regard to the functional overlap between environmental policies and the risk of deforestation; (ii) Second, at regional level for the states of Chiapas and Yucatan, we analyse the spatialization of the PAS-H during the 2005–2010 period in terms of key socio-economic criteria (e.g. deforestation risk and marginality) with regard to the role played by the changing political agenda and the structure of incentives from technical intermediaries.

The paper is organised as follows: Section 2 presents the conceptual framework and the case study. Section 3 presents the results as a combination of qualitative stakeholder and statistical analysis at national and regional level. Section 4 discusses the implications of the multi-level governance in shaping the policyscape with regard to the objective of tackling deforestation and poverty and the utility of the proposed framework to analyse the spatial outcomes of multi-level policy processes.

## Methods and Case Study

A multi-level governance analysis implies understanding how government and non-government organisations articulate their interests along the policy cycle of a program [14]. A classical policy cycle sequence includes: (i) The definition of goals and program design; (ii) the identification of funding resources; (iii) the implementation of the program; (iv) the evaluation of outputs; and (v) the evaluation of outcomes. The first three stages are directly determined by formal and informal governance networks, where voluble and informal negotiations and alliances take place [15,16]. The policy cycle is closed by a redefinition of policy goals through renewed institutional negotiation in a similar fashion as the adaptive management cycle [9,17].

**Fig 1:**
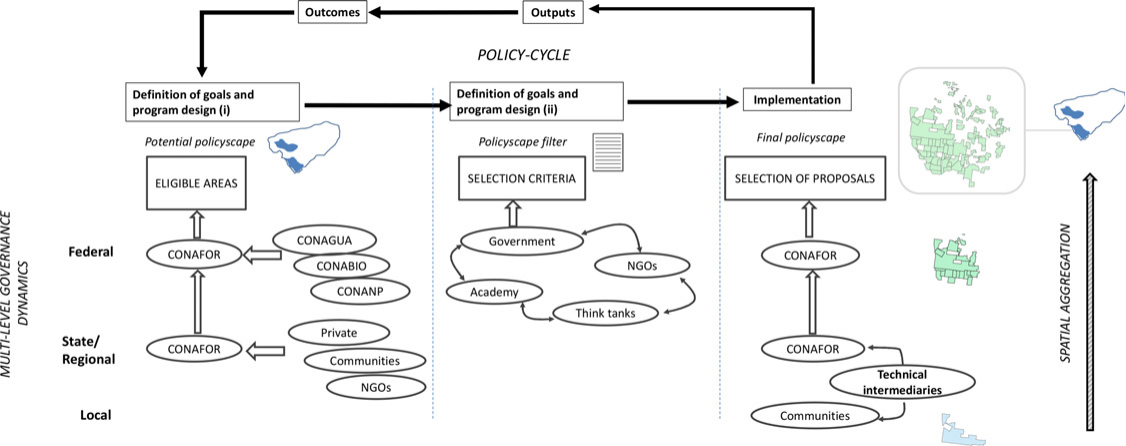
Analysis framework: Multi-level governance and policyscape dynamics in the PSA-H policy cycle.

The policy cycle of the Mexican PSA-H program is characterised by different governance spaces at federal and regional levels that coexist in a complex organisational architecture where a large number of actors and institutions interact. Such multi-level governance architecture makes possible the confluence of public and private actors that intervene at three different phases of program implementation: the definition of the eligible areas, the selection criteria for enrolling forest parcels, and the final selection of proposals (Fig 1). Eligible areas correspond to areas where forest communities can apply to the program. CONAFOR headquarters *draw* these areas using spatially explicit geo-physical (slope, vegetation, hydrological data, forest density) and socio-economic (presence of large cities, marginality, hydraulic infrastructures) data. Decision upon how final eligible areas will look like needs the formal approval of forest-related government bodies, such as the National Commissions for Water (CONAGUA), for Biodiversity (CONABIO) and for Natural Protected Areas (CONANP). Eligible areas are the spatial corset from which communities can potentially apply to the program. Then, forest parcels submitted to CONAFOR are classified based on a scoring system of selection criteria decided at federal level by a multi-stakeholder commission composed of public, private and civil society representatives [11]. After eligible areas and selection criteria are set, the program is launched in each state by CONAFOR offices, in charge of administrative procedures. Technical intermediaries (TI) are the ones to submit the PSA-H dossier and therefore assure the nexus between CONAFOR state offices and forest communities. Because they play a crucial role at enrolling communities, their decisions will influence the socio-economic and environmental characteristics of the areas where the PAS-H is implemented.

**Fig 2:**
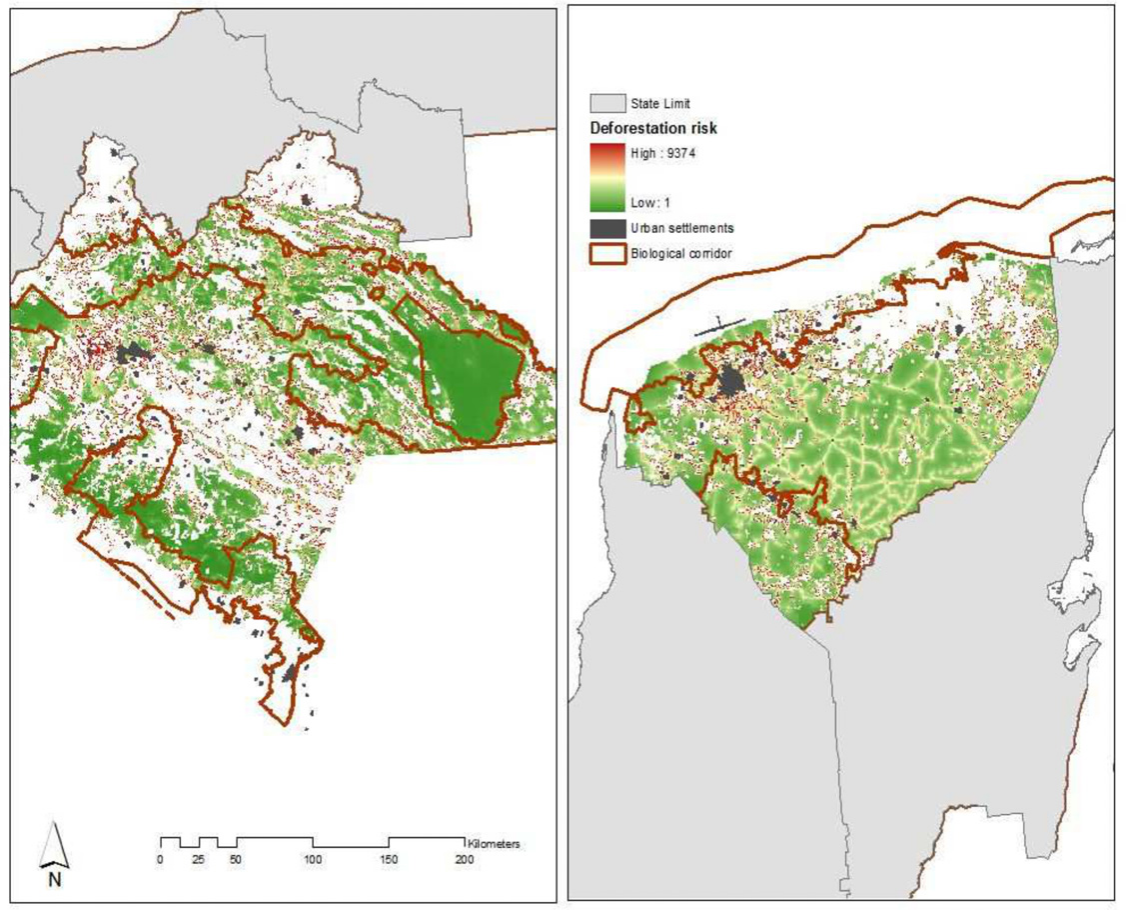
Forest cover and deforestation risk in the states of Yucatan and Chiapas. Sources: Institute of Statisitcs and Geography (INEGI) and Institute of the Ecology and Climate Change (INECC).

To understand the influence of the PSA-H multi-level governance on the resulting policyscape we use an analysis framework (Fig 1) that combines qualitative and quantitative research methods (Table 1). We apply semi-structured interviews for exploring stakeholder behaviour in two distinct situations: First, for the period ranging from 2004 to 2012, we explored the outcomes of the negotiation process in charge of designing the selection criteria. We reviewed existing evidence completed with semi-structured interviews of key competing actors. Second, we followed the changes in eligible areas in the states of Chiapas and Yucatan through interviews with federal and state CONAFOR staff and non-government organisations that had an active and influential role in the implementation of the program. The states of Chiapas and Yucatan are both characterized with large tracts of forests, contrasting ecological characteristics, where deforestation is high and forest communities are still abundant and tied to forest management (Fig 2). Moreover, in the state of Chiapas poverty is a main political issue and forest conservation has been strongly enforced through natural protected areas and biosphere reserves. In the flat state of Yucatan, forests are under the threat of cattle expansion and an expanding informal land market which both have been pushing up deforestation in the last decade [18,19]. Finally, we explore the motivations and structure of incentives of technical intermediaries during two expert workshops in the cities of Merida and Tuxtla-Gutiérrez (S1 Appendix).

**Table 1:** Summary of the data used in the study and corresponding analysis framework. Numbers in parenthesis indicate the number of participants per institution.

Data	National questionnaire of forest rural communities	Structured interviews with government and civil society	Yucatan and Chiapas spatially explicit database	Structured interviews and expert workshops (EW) in Yucatan and Chiapas
Year	2011	2012 and 2013	2012	2013
N	324	13	2681 for state-wide analysis	14 (Yucatan), 12 (Chiapas)
Coverage	National representativeness	Federal headquarters in Guadalajara and Mexico City	Regional: States of Yucatan and Chiapas	Mérida in Yucatan and Tuxtla-Gutiérrez in Chiapas
Nature of respondents by organization type	Board of rural communities	CONAFOR Guadalajara (6),INECC Mexico City (2),CONAGUA (1), CONANP (1), NGOs part of national PSA-H commission (4)(GAIA, Mexican Civil Council for Sustainable Forestry, the World Wildlife Fund, Pronatura)	NA	EW Mérida: CONAFOR regional (3)CONANP (1)CONAGUA (2)NGOs (3) (The Nature Conservancy, Niños y Crías, Pronatura)Technical intermediaries (6)EW Tuxtla: CONAFOR regional (2)Other gov. (CONANP) (1)NGOs (3) (Ambio, Pronatura, Conservation International), Technical intermediaries (5)
Dimensions measured	Location, participation in government programs	Role in designing the eligible areas and selection criteria	Participation in PSA-H, Payments for biodiversity, Protected Area. Socio-economic indicators including deforestation risk	Criteria for choosing communities
Analysis framework	Policyscape: Functional overlap	Governance analysis: Federal for the selection criteria and regional for the eligible areas	Policyscape: PAS-H spatialization	Governance analysis: Intermediaries

Descriptive statistical analyses are performed at two levels: At national level, we describe the functional overlap between environmental (including the PSA-H) and production support programs and the deforestation risk. We used data from the National Survey of Agrarian and Forestry Settlements of 2011 (ENNAF), a national survey previously performed in 2002 for a nationally representative random sample of 324 rural communities. Besides gathering data on the socio-economic characteristics and forest economic activities, the questionnaire gathers unique data on what programs are being implemented in each interviewed community. These programs fall into seven government programs: payments to support traditional maize agriculture (PROCAMPO), payments to support health-care costs of cattle (PROGAN), support to reforestation and soil restoration in forest areas (PROCOREF), support to forest management (PRODEFOR), payments for biodiversity (PSA-B), payments for hydrological services (PSA-H) and natural protected areas (NPAs). All forest programs (PRODEFOR, PROCOREF, PSA-H and PSA-B) are included under the umbrella of the ProArbol—Renamed as PRONAFOR in 2013- macro-program (S1 Table).

Then, at regional level for the states of Yucatan and Chiapas, we compiled a spatially explicit database of rural and forest communities by linking information from several secondary sources (S2 Appendix). The database includes information from a total of 2,681 forest communities, which corresponds to the total number of rural communities in Yucatan and Chiapas reported in the National Agrarian Register (RAN). The database contains variables such as the deforestation risk, the level of marginality, the presence of a National Protected Area (NPA), the vegetation type and land use reported by the National Institute of Statistics and Geography (INEGI), the presence of hydrological and biodiversity payments and general socio-economic characteristics such as total number of land holders.

## Results

### Defining the policyscape at federal level: The negotiation of the selection criteria and its implications for targeting the deforestation risk

McAffee and Shapiro-Garza [20] and Shapiro-Garza [5] explain how three competing political agendas have been clashing at federal level to influence the design of PES programs in Mexico. These agendas include rural social movements–represented by the social movement ¡Movimiento El Campo no Aguanta Mas! (MECNAM), NGOs (the World Wildlife Fund, The Nature Conservancy, national NGOs), the World Bank and the Mexican government. As a result of the confluence of these actors, Shapiro-Garza [5] describes the design of Mexican PES programs as a hybrid agenda between neoliberal objectives seeking the creation of markets for ecosystem services and various political compromises from the ruling party to reach a consensus with the civil society to support peasant communities through cash transfers. Embedded in such a multifaceted clash of interests from the very origin of the PSA-H creation, the policy process behind the design of the PSA-H selection criteria has also been characterized by a similar confrontation of agendas since it started in 2006. Indeed, during the first two years of the program (2004–2006) communities were enrolled if they were located within hydrological vulnerable areas, harbouring large tracts of forests, and located near or within a protected area. Proximity to cities was also taken into account. Given the lack of a proper scoring system to sort applicants, CONAFOR administration enrolled forest parcels by the order of arrival of submissions [11]. The design of a classification system based on added score of a set of selection criteria was intended to fix this problem and provide an economically efficient system [11]. In practice, the multi-stakeholder committee established at federal level to decide these citeria saw the confrontation of two main agendas: One agenda pushed for an economically efficient scoring system privileging hydrological vulnerability and deforestation risk criteria, while the other defended pro-social and pro-environment management indicators (e.g. existence of community conservation areas, community located in a biological corridor, high percent of forest cover). The need to accommodate all demands progressively increased the number of criteria used, passing from 9 criteria in 2006 to 26 criteria in 2010, and resulting in a net decline of the influence of hydrological and deforestation risk in the total score. On the contrary, social and environmental criteria won pre-eminence: The latter came to represent 80% of the total possible score in 2010 against 56% in 2006 [11].

What has been the impact of the negotiation process on the overlap between environmental policies and the deforestation risk? To answer this question we explore what policymixes exist in our ENNAF national sample of 324 forest communities. Programs analysed are PROGAN, PROCOREF, PROCAMPO, ProArbol (standing for all forest programs including PSA-H) and NPAs. To explore statistical association trends among programs we perform a principal component analysis (PCA) (Table 2). The three factors extracted express three different patterns: the first factor expresses the simultaneous application of forest management programs and PSA programs (F1); the second factor shows the coincidence between protected areas and PSA-H (F2); and the third factor associates PROGAN with reforestation activities and negatively associated with PSA-H (F3). We pursued our analysis by creating clusters of policymixes. Using a partition-clustering method based on the 3 factors of the PCA, we derived 3 categories of policymixes to which we added a de facto category created to include those communities with no other program but production support ones as PROCAMPO and PROGAN. The first group (C1) corresponds to a policymix where forest programs are strongly associated. This can be interpreted as both the reflection of the increased weight given to pro-forest selection criteria and the fact that technical intermediaries might allocate various forest programs to communities with large tracts of forests. The second group (C2) is a policymix characterised by communities in protected areas and with a strong presence of forest programs, in particular the PSA-H program. Again, this policymix is consistent with the increasing weight of environmental criteria in the selection criteria. The last two groups discriminate policymixes made of pure agricultural programs (C4)–support to cattle (PROGAN) and to traditional agriculture (PROCAMPO)- and (C3) PROGAN with some forest-related programs, in particular the reforestation program (PROCOREF).

**Table 2:** Policy-mixes defined by principal component analysis followed by a partition-clustering analysis. The three retained factors after rotation account for 66% of total variance. Scores in bold indicate PCA loadings higher than 0.3 or grouping frequencies bigger than 50%.

	Factor analysis			Cluster analysis			
Variables	F1ProArbol	F2NPA&PSA-H	F3PROGAN	C1ProArbol	C2 PA&PSA-H	C3PROGAN	C4Agr. only*
PROGAN	-0.098	0.059	**0.855**	49%	**72%**	**84%**	50%
PROCOREF	0.272	-0.067	**0.347**	**71%**	**59%**	**79%**	0%
PRODEFOR	**0.434**	-0.219	0.041	**72%**	28%	27%	0%
PSA-CABSA	**0.460**	-0.026	-0.077	**59%**	26%	6%	0%
PSA-H	**0.327**	**0.414**	-0.259	**67%**	**59%**	13%	0%
NPA	-0.126	**0.810**	0.101	0%	**100%**	0%	0%
% communities in categories				**28%**	**12%**	**33%**	**27%**

* Ag. only: only agriculture and cattle programs (PROCAMPO and PROGAN).

**Fig 3:**
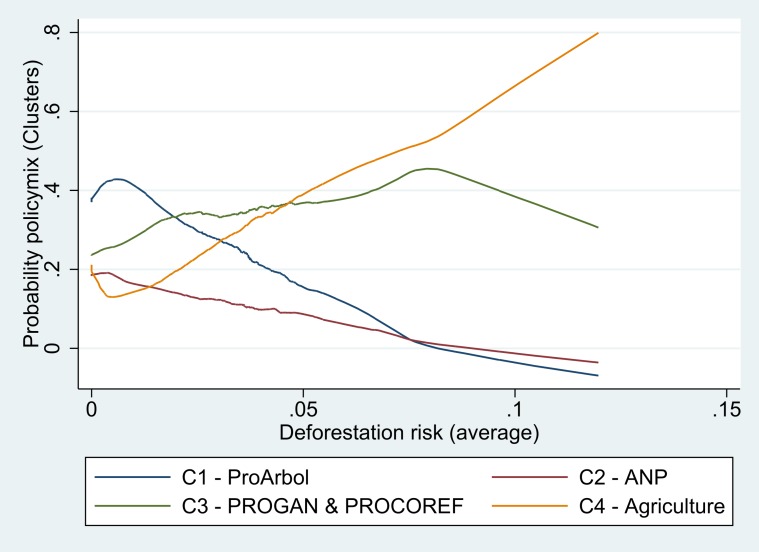
Functional overlap of environmental policymixes with the deforestation risk.

Next, we assess the functional overlap between the four identified groups of policymixes and the deforestation risk of forest communities by plotting the probability of finding each policymix with regard to an axis accounting for the deforestation risk (Fig 3). The probability of finding forest and environmental policymixes (C1 and C2) decreases with the increase of the deforestation risk. This decrease is sharper for the policymix (C1) grouping all forest programs including the PSA-H. On the contrary, the probability of finding agricultural programs (PROCAMPO with or without PROGAN) increases with the deforestation risk. Although no causality links can be derived from these descriptive statistics, it does indicates how programmes are spatially grouped since the deforestation risk is a variable spatially referenced. We observe the existence of three policyscapes: a first one where forest programs, the PSA-H and NPAs (C1 and C2) overlap in a zone of low deforestation risk; a second one where productive and reforestation programs (C3) work together regardless the deforestation risk and a third one where pure productive programs (C4) are associated with a high deforestation risk.

These patterns suggests that environmental policies, in close association with the PSA-H, are implemented in forests with low opportunity costs, where forest programs are economically attractive. This can be the result of both the failure of existing payment levels to attract forest communities with high deforestation opportunity costs and the result of governance dynamics that have prioritised social and environmental selection criteria, which correspond to forest communities with a low risk of deforestation.

### The spatialisation of the PSA-H at regional level: the cases of Chiapas and Yucatan

**Fig 4:**
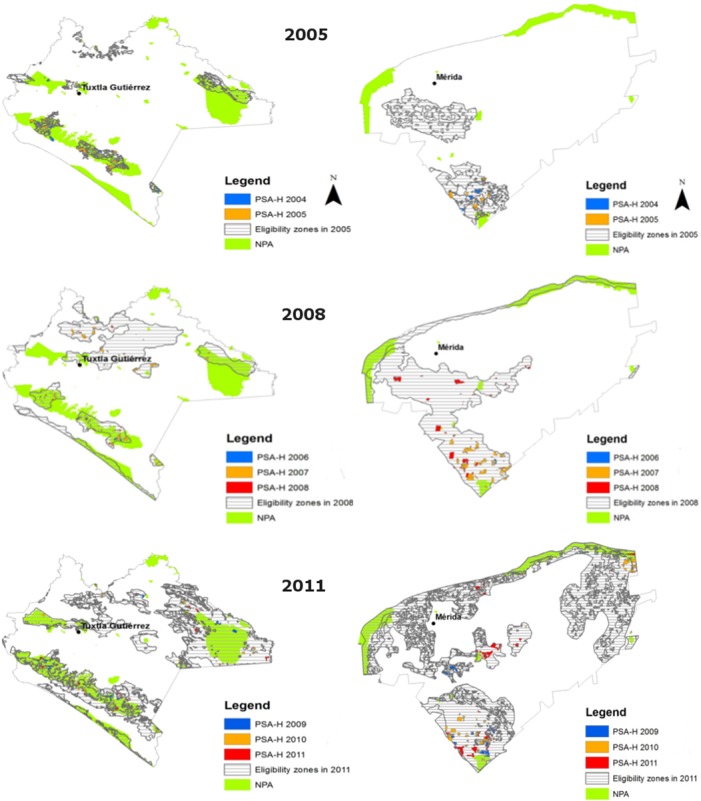
Evolution of PSA-HP eligible areas in Yucatan and Chiapas. Source: Authors with CONAFOR data.

PSA-H eligible areas have been in constant change during the 2004–2011 period as a result of fluctuating political agendas from different stakeholders. Fig 4 illustrates such changes for the states of Chiapas and Yucatan. The changes over time of the eligible areas respond to a governance process that experienced three different periods:

The first phase, during the years 2004 to 2006, correspond to the kick-off of the program. As part of a learning by doing process, in 2004 federal institutions set eligible areas to protect recharging areas of overexploited and vulnerable aquifers based on the presence of dense forest and on data from CONAGUA. Additionally, as part of the vertical governance process described in Fig 1, CONANP demanded that the program support communities in protected areas in order to compensate them for the costs of complying with conservation rules. Natural protected areas with dense forest cover where therefore included in the eligible areas. A final presidential criteria demanded that all states shall be eligible. The lack of overexploited aquifers in Chiapas and Yucatan meant protected areas in Chiapas and in the dense forests in the southern region of Yucatan became those areas the initially eligible areas. While social movements put pressure on CONAFOR to enlarge eligible areas in order to include neighbooring communities, the World Bank convinced CONAFOR officials to add areas nearby cities as they represented potential future service buyers (Fig 4 year 2005). In 2006, the eligible area remained unchanged. Overall, the delimitation of eligible areas during this first period responded to institutional sectorial objectives and the belief that the program would evolve towards a private market-based scheme.

During the second phase, from 2007 to 2009: In 2007, a major political change came into the scene. The newly elected president F. Calderón made the fight against poverty one of his political priorities, with an explicit reference to the support towards rural communities that assure the provision of forest ecosystem services to the country [21]. Such political committment provoked a sharp increase in CONAFOR budget and the inclusion of the marginality index as a key indicator to expand PSA-H eligible areas. As a result, the state of Chiapas experienced an expansion of the new eligible areas to regions with high marginality (Fig 4 year 2008). In addition, CONAFOR staff decided to compact eligible areas and fill gaps is order to facilitate administrative procedures. In 2008 CONAFOR included the demand from CONABIO to include mangroves and biodiversity corridors. This second period was characterised by an overall expansion of the eligible area pushed by the political will to include marginal rural communities.

The third phase, from the year 2010 to 2012 saw a major administrative change. In 2010, the biodiversity and hydrological payments for environmental services were officially merged into one program, divided in six spatially different regions: The first 3 ones with the highest payments were devoted to hydrological services, whereas the last 3 ones with smaller payments were under biodiversity services eligible areas (Fig 4 year 2011). CONAFOR assigned applications to one or another payment type depending on the geographical location of communities. This merging process responded to a budget decrease for biodiversity payments compared to the larger budget for hydrological services and from the pressure from NGOs and social movements that did not want the payments for biodiversity protection to disappear [5]. In 2011, CONAFOR decided to eliminate eligible areas that did not apply to the program in previous years in order to prioritise those that expressed their will to participate but were not eligible. The year 2012 was a political election year and the eligible areas remained unchanged.

How have these changes affected the spatialization of the program in terms of targeting forest at risk, poor populations and its association with other environmental programs such as NPAs? We compare the means of key socio-economic indicators from communities within and without the eligible areas from 2004 to 2010, the latter being the year when biodiversity and hydrological eligible areas were merged (Table 3, S2 Table). In the state of Chiapas, we find that differences between eligible and non-eligible areas are fairly stable across years. Eligible areas have been characterised since 2004 by communities with larger areas, within natural protected areas and with higher marginality and percentage of forest cover. Population density does not show important differences. Nevertheless, the deforestation risk scores significantly higher in communities outside eligible areas than within. The state of Yucatan shows a similar yet less noticeable pattern, with eligible forests being in larger communities with more forest, less populated and with a smaller risk of deforestation. Marginality shows a higher rate after the year 2010. An increase in the number of eligible communities occurs in 2007 when the targeting of marginal communities expands eligible areas.

**Table 3:** Difference in means for eligible vs non-eligible and PSA-H vs no PSA-H communities in the states of Chiapas and Yucatan.

				All state			Within eligible area		
				Eligible communities	Non eligible communities		Communities with PSA-H	Communities without PSA-H	
**CHIAPAS**									
	**N**			1166	784		131	1035	
	**Criteria**								
		risk deforestion		2.40	3.00	*	1.76	2.52	*
		% forested land		58%	31%	*	73%	56%	*
		marginality index		0.53	0.14	*	0.50	0.55	
		NPA		26%	10%	*	59%	23%	*
	**Community characteristics**								
		size (ha)		2198	1428	*	2806	2126	
		pop. density		0.51	0.88		0.35	0.53	
**YUCATAN**			** **						
	**N**			250	481		59	191	
	**Criteria**								
		risk deforestation		3.50	3.70	*	3.56	3.46	
		% forest		78%	77%		87%	87%	
		marginality		-0.09	0.01		0.37	-0.23	*
		NPA		17%	2%	*	17%	17%	
	**Community characteristics**								
		size (ha)		3978	2500	*	4827	3716	
	** **	pop. density		0.38	1.90		0.07	0.48	*

*: significant at 90% from 2004 to 2010.

However, eligible areas are just a filter for selecting communities. Enrolled communities need the assistance of technical intermediaries (TI) to participate in the program. In order to capture their possible role in defining the spatialization of the PSA-H, we assess the characterisitics of enrolled vs non-enrolled communities within eligible areas (Table 3, S3 Table). In the state of Chiapas, enrolled communities have more forest, were more frequently associated with natural protected areas and had lower deforestation risk than non-enrolled communities. These differences suggest that the establishment of eligible areas in Chiapas was particularly efficient at targeting marginal communities. The strong association of enrolled communities with low deforestation risk and larger tracts of forests suggests that technical intermediaries in Chiapas chose communities where the total amount of payments would be important–i.e. large tracts of forests- and presumably easy to be accepted by the assembly–i.e. low deforestation risk-. Moreover, the statistically significant highest frequency of NPAs in enrolled communities also reflects thepreference of technical intermediaries to target communities within or in the border of NPAs. TI have thus strengthened the preference of eligible areas to overlap with NPAs. In the state of Yucatan, enrolled communities have a lower population density and a higher marginal index. The percentage of the community area under forest is not statistically different, in line with the fact that eligible areas had already targeted densely forested areas. No difference in means were also found in the deforestation risk.

To crosscheck the statistical results we explored, during the two expert workshops with stakeholders, what structure of incentives guided the choice of TI with regard to what communities they would prefer to support. In Yucatan TI were mainly small consulting firms specialised in rural development. They expressed a preference to work with communities with large forests and fewer households. They also prioritise communities where governance is transparent, with a good leadership. Finally, they prefer to exclude communities where forest is under high deforestation pressure, near cities or affected by future infrastructure developments (e.g. roads, pipelines), since thay are aware that payments will not compete with potential gains from deforestation. In the state of Chiapas TI are composed of environmental and biodiversity conservation NGOs whose agenda is tied to supporting conservation and development activities in NPAs, areas where they have a working record.

Such behavior is in line with the objective of minimising contracting transaction costs (costs of gathering information, reaching and convincing the communities, administrative procedures for submitting the dossier, signature of the contract) [12] but also to limit uncertainty factors–as an unpredicted source of transaction costs, in line with the concept of bounded rationality [22]. From the intermediaries’ rationale of selecting forest communities we identify two major sources of uncertainty. A first source is associated with the reaction of communities after they are enrolled in the program and receive the first payment. The exclusion or inclusion of households within a community in formal internal agreements–making them eligible or not for payments- can create tensions and conflicts if the internal distribution of the payment is seen as unfair [23]. Working with a reduced number of households reduces the risk of conflicts and TI have also the capacity to monitor households and undertake a more accurate community governance diagnostic. In many cases communities under good governance are well known among neighbours and local institutions and in some cases are formally recognised through government certificates, such as CONANP certification for communities implementing voluntary conservation reserves (http://www.conanp.gob.mx/difusion/comunicado.php?id_subcontenido=75). Working in areas where intermediaires have a working record–i.e. in NPAs- also disminishes this source of uncertainty while being able to adopt a long term support strategy. A second source of uncertainty is related to the mistrust towards the capacity of the administration to assure funds for the whole 5-year cycle of payments. TI will prefer selecting communities with large forests in order to get larger payments and bigger commissions for their services.

## Discussion

National government PES programs entail large and complex governance structures involving multiple sequential implementation steps at different geographic scales. For policy targeting to assure program objectives, the multi-level governance has to be aligned to program objectives all along the policy process [24]. The Mexican PSA-H program is a good project-specific case study to assess the impact of the policy process in targeting outcomes. At the federal level, the probability of finding environmental policymixes along a deforestation risk axis shows a clear spatial segmentation between agricultural and forest programs, the latter negatively associated with the deforestation risk. Such segmentation reflects both the evident matching of programs with land use profitability (and therefore opportunity costs) and the effect of governance dynamics that shaped the design of the selection criteria. This interpretation, nevertheless, must be taken with cautioun since the national sample we use is relatively small (324 communities) and only for the year 2011. A more robust assessment would benefit from panel data in order to assess the concordance between the evolution of selection criteria, policymixes and the deforestation risk on a yearly basis.

The selection criteria operates as a filter within the eligible areas to enroll best-suited communities. The eligible area, settled at federal level after consulting different government and non-government stakeholders, increased along the years as a result of the integration of more objectives–in a similar manner as the design of the selection criteria- and a sharp budget increase under the political will of the F. Calderon presidency. Interestingly, the mean values of key socio-economic criteria in eligible areas remained fairly similar across the yearswhich suggests a resilient eligibility strategy from the federal forest administration since its implementation. Nevertheless, the spatialization of the PSA-H emerges only after technical intermediaries come into action. Existing theorethical models on the influence of technical intermediaries of the effectiveness of PES point out that they select communities mainly based on the minimisation of transaction costs and sources of uncertainty [12,22]. By targeting communities with low opportunity costs, large tracts of forests (i.e. in marginal geographic areas) and which are not densely populated, TI have strengthened the underlying targeting pattern arising from the designed eligible areas with only small modifications in the type of communities enrolled. This result can be explained as a combination of two factors. First, the choice of eligible areas seem to have coped well with institutional objectives, in terms of reinforcing protected areas, large forests and more marginalised communities. Forests with high deforestation risks have been systematically left aside. Second, the selection criteria and the standard assessment for allocating money to communities–based on the total number of hectares inscribed- is aligned with having low transaction costs (i.e. increasing the cost-effectiveness of their support) while minimising sources of uncertainty. At a qualitative level, we also observed that TI that have kept the fidelity of communities over the years have proven to have a long term vision of rural development. Indeed, in the early cohorts of the PSA-H, some TI tricked communities asking for more money or more payments than those expressed in CONAFOR implementation rules. A complementary study conducted by the authors in 77 communities in Southern Yucatan revealed that TI used to get paid on a yearly basis an average of 11% of the payment received by communities [23]. With the passing of time, only TI that have built long lasting trust with communities have been able to endure in the sector. As a result, they have also acquired a prospective vision of what development and conservation trajectories are needed for each community, and what programs are best suited. A quantitative analysis explaining how the structure of incentives of TI predicts communities characteristics and environmental outcomes would be needed to strengthen these first results. Moreover, since programs interact among themselves and with conditions of the ground driven by governance dynamics, our study proposes a methodology to deal with such complexity. Forthcoming studies in Mexico need to combine qualitative and quantitative spatially explicit datasets on ongoing productive and conservation programs in order to disentangle with more detail the causality links between these different processes.

## Conclusion

Large public PES programs rely on targeting to assure that political and institutional objectives are met. In the present paper we assess the impact of the multi-level governance of the Mexican program of payments for hydrological services on the targeting process for enrolling communities with high deforestation risk and marginality as the main two policy objectives. We combine the policyscape framework with a governance analysis at national and regional level for the states of Chiapas and Yucatan. At national level, PSA-H implementation can’t cope with high deforestation risk areas where land uses are specialized towards agricultural production. As a consequence, PSA-H is implemented together with and reinforcing already existing conservation programs like NPAs, and forest management programs. At regional level in the states of Yucatan and Chiapas, we explore the socio-economic characteristics of communities within eligible and enrolled areas. In both states, both eligible and enrolled communities fall in areas with extensive forests and associated with low deforestation risk. In Chiapas, eligible and enrolled areas include also high marginal zones, as a result of a political will. Finally, technical intermediaries seek to maximize profits while minimizing sources of uncertainty: They prioritize communities with large forests, reinforcing this specific feature of eligible areas, but also communities less densely populated and with good governance, in order to minimize negotiations costs and uncertainty with respect to the behavior of contracted parties. The combination of a quantitative policyscape framework with multi-level governance analysis is a useful tool to clarify the complex interactions between governance dynamics and policy spatial implementation outcomes in order to assess the degree of syncronisation between governance dynamics, policy objectives and landscape characteristics.

## Supporting Information

S1 Appendix Description of qualitative data and methods. (DOCX)

S2 Appendix Description of spatially explicit database. (DOCX)

S1 Table Policymixes from national sample of communities. (DOCX)

S2 Table Difference in means for eligible vs non-eligible communities in the states of Chiapas and Yucatan. (DOCX)

S3 Table Difference in means for enrolled vs non-enrolled communities in the states of Chiapas and Yucatan. (DOCX)
