# Supplementary material for: Payments for Environmental Services in a Policymix: Spatial and Temporal Articulation in Mexico
Source: PLoS One. 2016 Apr 6;11(4):e0152514. doi: 10.1371/journal.pone.0152514 (PMC4822810; doi:10.1371/journal.pone.0152514)
Supplement: S1 Appendix — (DOCX) [file pone.0152514.s001.docx]

**S1 Appendix. Description of qualitative data and methods.**

The analysis of the multi-level governance policy process for PSAH and factors influencing the changes in the policyscape was based on a combination of synthesis of existing research and original data gathering from interviews to different actors. For the selection of criteria at federal level we crosschecked existing literature with the description from involved stakeholders of the agendas involved in the negotiation and the tipping points regarding the relationships between actors. Sanginés et al. (2012) and Ezzine-de-Blas (2013) conducted semi-structure interviews with federal and state level Mexico’s National Commission of Forests (CONAFOR) administrators, NGOs with a leading role in the negotiation process (e.g. the Mexican Civil Council for Sustainable Forestry (CCMSS)) and other government structures pushing a particular agenda, such as the National Institute of Ecology and Climate Change (INECC) that defended the prioritization of the deforestation risk as the main selection criteria.

The semi-structured interviews focused on two aspects: The first objective was to understand the role of each institution in the process, the underlying alliances and their perception on the negotiation position of other organization and opposing alliances. The second objective was to understand the underlying reasons explaining the changes in eligible areas in the states of Chiapas and Yucatan. In total, 13 semi-structured interviews were conducted in between 2011 and 2013: 6 interviews were conducted in the federal headquarters of CONAFOR in the city of Guadalajara, capital of the state of Jalisco. These 6 interviews accounted for; (1) the general secretary of CONAFOR at the time of the F. Calderon administration; (1) the director of the division of Forest Productivity; (1) the director of the PSA-H sub-division; (1) the director of the department of valuation of forest ecosystem services and; (2) two staff in charge of GIS procedures. In Mexico City 7 more interviews were conducted. They account for: (2) two executive directors of INECC; (5) three representatives of non-government stakeholders, CCMSS, Iberoamericana University and the Mexican Forestry Fund and; (2) one representative of the Mexican Water Commission (CONAGUA) and one representative from the Mexican Commission for Natural Protected Areas (CONANP). Finally, for the states of Yucatan and Chiapas, in order to crosscheck the information gathered at federal level on the changes in eligible areas, we conducted (5) five additional interviews in the state of Yucatan and (4) four in the state of Chiapas. In the state of Yucatan the additional interviews account for (2) two interviews with CONAFOR regional executive director and assistant in the city of Merida and (3) interviews with non-governmental actors having a leading role in the implementation of the PSAH and in close interaction with CONAFOR state offices. These (3) interviews account for: the Yucatan office of The Nature Conservancy (TNC), and two local NGOs (Pronatura Yucatan and Niños y Crias). In the state of Chiapas, (4) four interviews were conducted with the CONAFOR regional executive director in the city of Tuxtla-Gutiérrez and (3) three interviews with local NGOS (AMBIO, Pronatura and, Conservation Fund for the Reserve of el Triunfo).

In addition, we conducted two expert workshops (EW) in the cities of Tuxtla-Gutiérrez and Mérida in May 2014 with technical intermediaries (TI) of the states of Chiapas and Yucatan. Both workshops were structured as follows: (i) an initiating session were researchers presented preliminary results changes in selection criteria and eligible areas in order to stimulate a free discussion; and (ii) a following session were working groups were created to discuss rationale guiding TI communities’ choices and what changes would be needed to improve program targeting and rural development including payments for environmental services. Besides a series of individual semi-structured interviews with TI that had assisted to the EW and other TI that had not assisted but were willing to be interviewed. The interviews were used to cross-check the EW main findings and to further understand the economical and motivational rationale for the selection of communities to be enrolled in the PSAH and the programs offered. A total of 14 attendants came to the EW in Mérida among which 6 TI. In Tuxtla-Gutiérrez 12 attended among which 5 TI.

**Additional references:**

Ezzine-de-Blas, D., Lara Pulido, J.A., Dutilly-Diane, C., LeVelly, G., Muñoz-Pina, C., Guevara Sanginés, A. 2013. Payment for Environmental Services in a policy-mix: Spatial and temporal articulation Mexico. Ecological Economics International Congress. L’Ille, France, 19-21 June 2013.

Guevara Sanginés, A. and Lara-Pulido, J.A. 2012. Evolución de los programas de la CONAFOR: Origen, desarrollo y perspectivas a futuro. CONAFOR, México D.F.
