# Supplementary material for: Payments for Environmental Services in a Policymix: Spatial and Temporal Articulation in Mexico
Source: PLoS One. 2016 Apr 6;11(4):e0152514. doi: 10.1371/journal.pone.0152514 (PMC4822810; doi:10.1371/journal.pone.0152514)
Supplement: S2 Appendix — (DOCX) [file pone.0152514.s002.docx]

**S2 Appendix. Description of the spatially explicit database.**

We build a spatially explicit database (available upon demand) with official information from 2,681 rural communities in the states of Chiapas and Yucatan. This database contains the polygons with the limits of the communities that are registered in the National Agrarian Registry (RAN). Additionally, we overlapped spatially explicit variables accounting for the following data: General descriptors (6), geomorphology (2), application and enrollment in PSAH across 2004 to 2010 (14), application and enrollment in PSACABSA across 2004 to 2010 (14), Participation in agricultural program (PROCAMPO), part of an PSAH eligibility zone from 2004 to 2010 (7), part of an natural protected area (7), deforestation risk estimated for 2010, marginality index for 2010, forests and pastures (14), other PSAH selection criteria (28).

For accessing the database please send an email to corresponding author: [ezzine@cirad.fr](mailto:ezzine@cirad.fr).
