# Supplementary material for: Payments for Environmental Services in a Policymix: Spatial and Temporal Articulation in Mexico
Source: PLoS One. 2016 Apr 6;11(4):e0152514. doi: 10.1371/journal.pone.0152514 (PMC4822810; doi:10.1371/journal.pone.0152514)
Supplement: S1 Table — (DOCX) [file pone.0152514.s003.docx]

**S1 Table. Policymixes from national sample of communities.**

The combination of programs from the 324 communities interviewed at a national level results in a total of 33 policymixes from a total maximum number of 57 possible combinations. The first three policymixes account for 44% of the 324 communities. Program names account for: Support to reforestation and soil restoration (PROCOREF); support to forest management (PRODEFOR); payments for biodiversity and carbon (PSA-CABSA); program maintain traditional agriculture (PROCAMPO), payments to support cattle (PROGAN); payments for hydrological services (PSAH).

| Policymix # | PROCOREF | PRODEFOR | PSACABSA | PROCAMPO | PROGAN | PSAH | Cumulative frequency |
| --- | --- | --- | --- | --- | --- | --- | --- |
| 1 |  |  |  | • | • |  | 16% |
| 2 |  |  |  | • |  |  | 30% |
| 3 | • |  |  | • | • |  | 44% |
| 4 | • | • |  | • | • |  | 49% |
| 5 | • |  |  | • |  |  | 54% |
| 6 | • | • | • | • | • | • | 59% |
| 7 |  | • |  | • | • |  | 62% |
| 8 | • | • | • | • | • |  | 66% |
| 9 |  |  |  | • | • | • | 69% |
| 10 | • |  |  | • | • | • | 72% |
| 11 | • | • |  | • | • | • | 75% |
| 12 | • |  | • | • | • |  | 77% |
| 13 | • |  |  | • |  | • | 80% |
| 14 | • | • |  | • |  |  | 82% |
| 15 | • |  | • | • | • | • | 84% |
| 16 | • | • | • | • |  | • | 86% |
| 17 |  |  |  | • |  | • | 88% |
| 18 | • |  | • | • |  | • | 89% |
| 19 |  | • |  | • | • | • | 91% |
| 20 | • | • |  | • |  | • | 92% |
| 21 |  | • | • | • | • | • | 93% |
| 22 |  | • |  | • |  |  | 94% |
| 23 | • | • | • | • |  |  | 95% |
| 24 |  | • |  | • |  | • | 96% |
| 25 |  |  | • | • |  |  | 97% |
| 26 |  |  | • | • | • | • | 98% |
| 27 |  | • | • | • |  | • | 98% |
| 28 |  | • | • | • |  |  | 98% |
| 29 |  | • | • |  |  | • | 99% |
| 30 |  |  | • | • | • |  | 99% |
| 32 |  |  | • | • |  | • | 99% |
| 33 |  |  |  |  |  | • | 100% |
