# Supplementary material for: Payments for Environmental Services in a Policymix: Spatial and Temporal Articulation in Mexico
Source: PLoS One. 2016 Apr 6;11(4):e0152514. doi: 10.1371/journal.pone.0152514 (PMC4822810; doi:10.1371/journal.pone.0152514)
Supplement: S2 Table — (DOCX) [file pone.0152514.s004.docx]

**S2 Table. Difference in means for eligible vs non-eligible communities in the states of Chiapas and Yucatan.**

| Eligible vs. non-eligible | | 2004 | 2005 | 2006 | 2007 | 2008 | 2009 | 2010 |  | Average in* | Average out |
| --- | --- | --- | --- | --- | --- | --- | --- | --- | --- | --- | --- |
| Chiapas | | |  |  |  |  |  |  |  |  |  |
| Number of eligible  communities | | |  |  |  |  |  |  |  |  |  |
|  |  | 148 | 203 | 203 | 538 | 593 | 543 | 1 081 |  | 1166 | 784 |
| Community characteristics | | |  |  |  |  |  |  |  |  |  |
|  | size (ha) | . | + | + | +++ | +++ | +++ | +++ |  | 2198 | 1428 |
|  | pop. density | - | . | . | . | . | . | --- |  | 0.51 | 0.88 |
| Criteria | |  |  |  |  |  |  |  |  |  |  |
|  | marginality index | +++ | . | . | +++ | +++ | +++ | +++ |  | 0.53 | 0.14 |
|  | % forested | +++ | +++ | +++ | +++ | +++ | +++ | +++ |  | 58% | 31% |
|  | deforestion risk | --- | -- | -- | --- | --- | --- | -- |  | 2.40 | 3.00 |
|  | NPA | +++ | +++ | +++ | +++ | +++ | +++ | +++ |  | 26% | 10% |
| Yucatan | | |  |  |  |  |  |  |  |  |  |
| Number of eligible  communities | | | |  |  |  |  |  |  |  |  |
|  |  | 61 | 144 | 144 | 159 | 231 | 205 | 136 |  | 250 | 481 |
| Community characteristics | | |  |  |  |  |  |  |  |  |  |
|  | size (ha) | . | + | + | +++ | +++ | +++ | +++ |  | 3978 | 2500 |
|  | pop. density | . | . | . | . | - | - | . |  | 0.38 | 1.90 |
| Criteria | |  |  |  |  |  |  |  |  |  |  |
|  | marginality index | + | . | . | . | - | - | +++ |  | -0.09 | 0.01 |
|  | % forested land | + | + | + | + | . | . | + |  | 78% | 77% |
|  | deforestion risk | . | - | - | - | - | - | . |  | 3.50 | 3.70 |
|  | NPA | . | . | . | + | +++ | +++ | +++ |  | 17% | 2% |

+/- significant at 90%; ++/-- significant at 95%; +++/--- significant at 99%

*Average in = average for ejidos that enrolled at least one time in the 2004-2010 period

Evolution of the difference in means between eligible and non-eligible communities in the states of Chiapas and Yucatan (annual indicators and average indicators for the period 2004-2010). ‘+’ sign indicates a positive difference in means. ‘-‘ sign indicates a negative difference. ‘.’ Sign indicates no statistical significant difference.
