# Supplementary material for: Payments for Environmental Services in a Policymix: Spatial and Temporal Articulation in Mexico
Source: PLoS One. 2016 Apr 6;11(4):e0152514. doi: 10.1371/journal.pone.0152514 (PMC4822810; doi:10.1371/journal.pone.0152514)
Supplement: S3 Table — (DOCX) [file pone.0152514.s005.docx]

**S3 Table. Difference in means for enrolled vs non-enrolled communities in the states of Chiapas and Yucatan.**

|  | | |  | |  | |  | |  | |  | |  | |  | |  | |  | | Average with | |  | | Average without | |
| --- | --- | --- | --- | --- | --- | --- | --- | --- | --- | --- | --- | --- | --- | --- | --- | --- | --- | --- | --- | --- | --- | --- | --- | --- | --- | --- |
| PSA-H vs. non PSA-H | | | | | 2004 | | 2005 | | 2006 | | 2007 | | 2008 | | 2009 | | 2010 | |  | |  |  |  | |  |  |
| Chiapas | | | | | | |  | |  | |  | |  | |  | |  | |  | |  | |  | |  | |
| Number of new enrolled  communities | | | | | | | | |  | |  | |  | |  | |  | |  | |  | |  | |  | |
|  | | |  | | 13 | | 30 | | 4 | | 47 | | 14 | | 15 | | 29 | |  | | 131 | |  | | 1035 | |
| Community characteristics | | | | | | |  | |  | |  | |  | |  | |  | |  | |  | |  | |  | |
|  | | | size (ha) | | . | | . | | . | | . | | . | | . | | . | |  | | 2806 | |  | | 2126 | |
|  | | | pop. density | | . | | . | | . | | . | | . | | . | | . | |  | | 0.35 | |  | | 0.53 | |
| Criteria | | | | |  | |  | |  | |  | |  | |  | |  | |  | |  | |  | |  | |
|  | | | marginality index | | - | | . | | . | | . | | - | | - | | . | |  | | 0.50 | |  | | 0.55 | |
|  | | | % forested | | . | | ++ | | ++ | | ++ | | +++ | | +++ | | ++ | |  | | 73% | |  | | 56% | |
|  | | | deforestion risk | | --- | | -- | | -- | | -- | | . | | -- | | -- | |  | | 1.76 | |  | | 2.52 | |
|  | | | NPA | | + | | +++ | | +++ | | +++ | | +++ | | +++ | | +++ | |  | | 59% | |  | | 23% | |
| Yucatan | | | | | | |  | |  | |  | |  | |  | |  | |  | |  | |  | |  | |
| Number of new communities contracted | | | | | | |  | |  | |  | |  | |  | |  | |  | |  | |  | |  |  |
|  |  | | 3 | | 6 | | 0 | | 22 | | 15 | | 9 | | 19 | |  | | 59 | |  | | 191 | |  |  |
| Community characteristics | | | | |  | |  | |  | |  | |  | |  | |  | |  | |  | |  | |  |  |
|  | size | | . | | . | |  | | . | | +++ | | . | | . | |  | | 4827 | |  | | 3716 | |  |  |
|  | popdens | | . | | . | |  | | --- | | -- | | -- | | -- | |  | | 0.07 | |  | | 0.48 | |  |  |
| Criteria | | |  | |  | |  | |  | |  | |  | |  | |  | |  | |  | |  | |  |  |
|  | marginality | | + | | + | |  | | . | | ++ | | ++ | | ++ | |  | | 0.37 | |  | | -0.23 | |  |  |
|  | % forest | | . | | . | |  | | . | | . | | . | | . | |  | | 87% | |  | | 87% | |  |  |
|  | deforest risk | | . | | . | |  | | . | | . | | . | | + | |  | | 3.56 | |  | | 3.46 | |  |  |
|  | NPA | | . | | . | |  | | . | | . | | . | | . | |  | | 17% | |  | | 17% | |  |  |

+/- significant at 90%; ++/-- significant at 95%; +++/--- significant at 99%

*Average in = average for ejidos that enrolled at least one time in the 2004-2010 period

Evolution of the difference in means between enrolled and non-enrolled communities in the states of Chiapas and Yucatan (annual indicators and average indicators for the period 2004-2010). ‘+’ sign indicates a positive difference in means. ‘-‘ sign indicates a negative difference. ‘.’ sign indicates no statistical significant difference.
